# Epidemiology of gout in Hong Kong: a population-based study from 2006 to 2016

**DOI:** 10.1186/s13075-020-02299-5

**Published:** 2020-09-04

**Authors:** Man Fung Tsoi, Man Ho Chung, Bernard Man Yung Cheung, Chak Sing Lau, Tommy Tsang Cheung

**Affiliations:** 1 Department of Medicine, The University of Hong Kong, Pokfulam, Hong Kong China; 2 Department of Medicine, Queen Mary Hospital, Pokfulam, Hong Kong China; 3 Partner State Key Laboratory of Pharmaceutical Biotechnology, The University of Hong Kong, Pokfulam, Hong Kong China; 4 Research Centre of Heart Brain, Hormone and Healthy Aging, The University of Hong Kong, Pokfulam, Hong Kong China; 5 Department of Medicine, Hong Kong Sanatorium & Hospital, 2 Village Road, Happy Valley, Hong Kong China

**Keywords:** Gout, Hong Kong, Epidemiology, Urate-lowering therapy

## Abstract

**Objective:**

To determine the incidence and prevalence of gout in the general population and the utilisation of urate-lowering therapy (ULT) among patients with gout in Hong Kong.

**Methods:**

A total of 2,741,862 subjects who attended any outpatient clinics or accident and emergency department (with or without hospitalisation) in 2005 and did not die before 2006 were identified from the Clinical Data Analysis and Reporting System (CDARS) of the Hospital Authority in Hong Kong. All subjects were followed until the end of 2016 or death.

Demographics, diagnosis of gout, serum urate levels, and ULT prescriptions were retrieved from CDARS. Gout was defined by the diagnosis codes in CDARS. The serum urate levels achieved after prescribing ULT were the means of all serum urate levels measured 6 months after prescriptions. Results were analysed by R version 3.3.3 with package ‘prevalence’ version 0.4.0.

**Results:**

The crude incidence of gout increased from 113.05/100,000 person-years (PY) in 2006 to 211.62/100,000 PY in 2016. The crude prevalence of gout increased from 1.56% in 2006 to 2.92% in 2016. Only 25.55% of patients with gout were prescribed ULT in 2016. 35.8% of patients treated with ULT were able to achieve the target serum urate level of < 6 mg/dL.

**Conclusions:**

Population ageing as well as other risk factors contributed to an increase in the incidence and prevalence of gout in Hong Kong. In 2016, the crude prevalence of gout in Hong Kong was comparable to that in many western countries. However, only one in four patients with gout in Hong Kong was prescribed ULT.

## Introduction

Gout is the most common inflammatory arthritis caused by deposition of monosodium urate crystals in peripheral joints. Although the prevalence of gout was estimated to be 0.08% globally [1], it has increased significantly over the last decade, especially in developed countries and Oceanic populations. The estimated prevalence of gout in the United States (US), UK, and European countries is 2–3% [2, 3]. Asian countries and regions except Taiwan are considered to have a lower prevalence of gout due to differences in ethnicity and lifestyle [4, 5].

The American College of Rheumatology (ACR) and European League Against Rheumatism (EULAR) recommend urate-lowering therapy (ULT) in all patients with recurrent flare, tophi, chronic kidney disease, and urolithiasis. In particular, EULAR also recommends initiation of ULT close to the time of first diagnosis in patients with early-onset gout or with a very high baseline serum urate level. The target serum urate level should be maintained below 6 mg/dL but not less than 3 mg/dL in the long-term [6, 7].

A local retrospective study of 279 subjects reported that 70% of patients with gout were prescribed ULT. However, the study results could not be generalised to the population because it was conducted in a family medicine training centre [8]. Due to the lack of latest epidemiological data on gout in Hong Kong, we conducted this population-based study to determine the incidence and prevalence of gout as well as the utilisation of ULT among patients with gout in Hong Kong.

## Methods

The Hospital Authority (HA) is the only public healthcare provider in Hong Kong. More than 90% of Hong Kong citizens utilise the public healthcare service [9]. The Clinical Data Analysis and Reporting System (CDARS) is a database managed and updated daily by the HA [10]. This database captures clinical parameters of each public healthcare service user. These parameters include patient demographics, death, diagnoses, hospital admissions, procedures performed, laboratory parameters, medication prescriptions, and dispensing histories. It has been used to conduct high-quality epidemiological studies of infectious and rheumatic diseases [9, 11].

We included all subjects who attended any outpatient clinics or accident and emergency department (with or without hospitalisation) in 2005. Since all the data are anonymised in CDARS, each user was assigned a unique reference key and it was used to identify duplicate records in different datasets, i.e. outpatient clinics and accident and emergency department visits. Subjects who died before 1 January 2006 were excluded. Clinical data including demographics, diagnosis of gout, serum urate levels, and prescriptions of ULT were retrieved from CDARS from 1 January 2006 to 31 December 2016.

All subjects were followed until the cut-off date (31 December 2016) or death. Gout was defined as physician-diagnosed gout according to the International Classification of Diseases 9th Edition diagnosis codes. These diagnosis codes include gouty arthropathy (274.0), gouty nephropathy (274.1), gouty tophi of the ear (274.81), gout with other specified manifestations (274.89), and unspecified gout (274.9). Validation of diagnosis was performed by a certified rheumatologist. This was conducted by accessing 500 electronic records of patients with gout and the same number of electronic records of patients without gout from Queen Mary Hospital. A positive predictive value of 97.5% and negative predictive value of 98.7% were demonstrated.

We defined the incidence of gout as the number of incident cases divided by the number of person-years (PY). The prevalence of gout was defined as the number of prevalent cases divided by the number of people included at the end of each calendar year.

The prescription of ULT was defined as a continuous prescription of ULT for more than 30 days. Benzbromarone, sulfinpyrazone, and peticoglycase were not included because they were not available in the HA drug formulary [12]. Therefore, we only estimated the percentages of patients prescribed allopurinol, febuxostat, or probenecid. In patients treated with ULT, only the serum urate levels measured 6 months after commencement of ULT were included for analysis. Mean serum urate levels and percentages of patients achieving the treatment target were stratified by sex and the use of ULT.

Results were analysed by R version 3.3.3 (https://www.R-project.org/) with package ‘prevalence’ version 0.4.0. Incidence rate, prevalence, and their 95% confidence intervals (CIs) were estimated. Age- and sex-adjusted incidence and the prevalence of gout were adjusted based on the population data released by the Census and Statistics Department, The Hong Kong Special Administrative Region Government [13]. We used population data in 2006 as the reference population in this study. Age- and sex-specific incidence and prevalence, and male-to-female incidence rate ratio and prevalence ratio were also estimated.

This study was approved by the Institutional Review Board of the University of Hong Kong/Hospital Authority Hong Kong West Cluster (IRB reference number: UW-533).

## Results

**Fig. 1:**
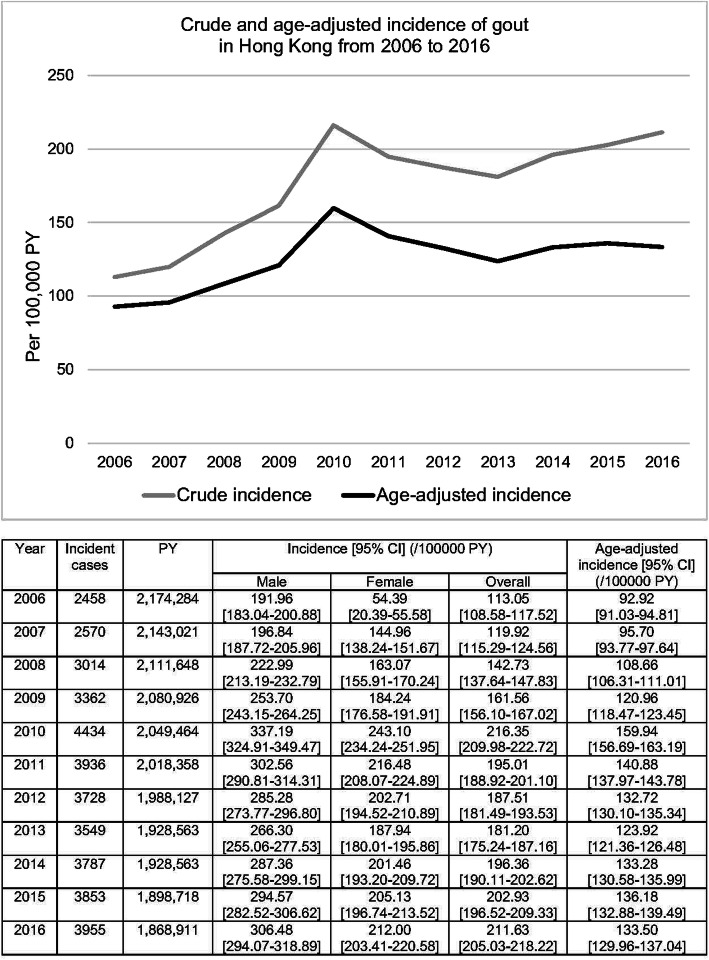
Incidence of gout in Hong Kong from 2006 to 2016

A total of 4,864,094 patients were identified in CDARS in 2005. After excluding 2,052,702 duplicate records and 69,530 deaths in 2005, 2,741,862 patients were included in the analysis. A summary of subject inclusion is summarised in Supplementary Fig. S1. The crude incidence [95% CI] of gout increased from 113.05 [108.58–117.52]/100,000 PY in 2006 to 211.62 [205.03–218.22]/100,000 PY in 2016. The age-adjusted incidence of gout showed an increase from 92.92 [91.03–94.81]/100,000 PY in 2006 to 112.52 [110.05–115.00]/100,000 PY in 2016 (Fig. 1). There was also a steady increase in the incidence of gout in both sexes. The age- and sex-adjusted incidence of gout tripled from 99.41 [97.70–101.12]/100,000 PY in 2006 to 294.74 [285.67–303.80]/100,000 PY in 2016 (Supplementary Table S1).

**Fig. 2:**
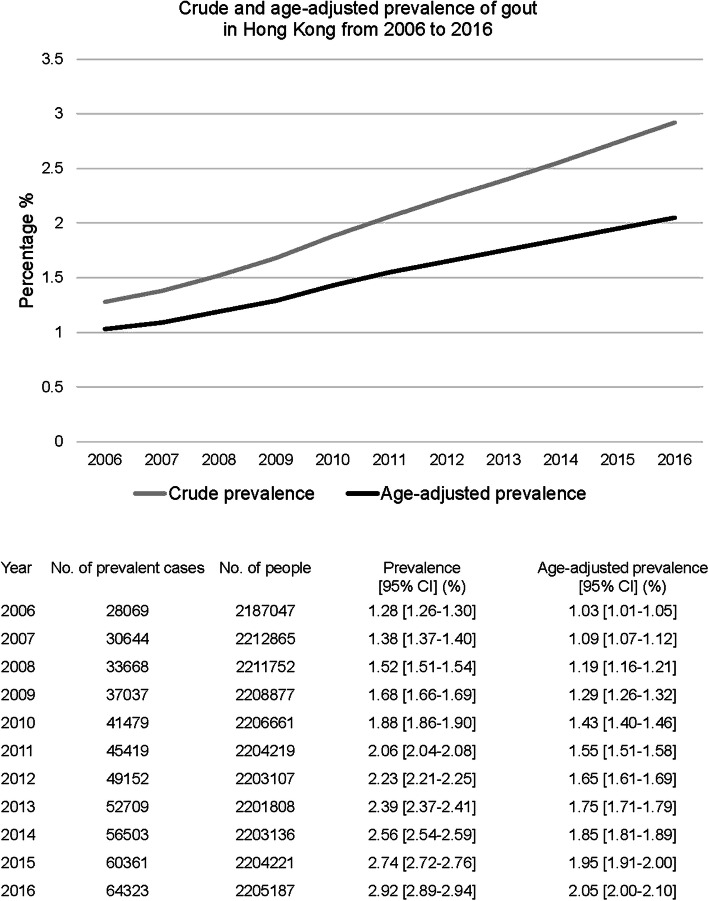
Prevalence of gout in Hong Kong from 2006 to 2016

The crude prevalence [95% CI] of gout increased from 1.28 [1.26–1.30] % in 2006 to 2.92 [2.89–2.94] % in 2016. The age-adjusted prevalence of gout increased from 1.03 [1.01–1.05] % in 2006 to 2.05 [2.00–2.10] % in 2016 (Fig. 2). The age- and sex-adjusted prevalence of gout in 2006–2016 in Hong Kong is summarised in Supplementary Table S1. The age- and sex-adjusted prevalence of gout doubled from 1.08 [1.06–1.10] % in 2006 to 1.84 [1.81–1.86] % in 2016.

The age- and sex-specific incidence and prevalence of gout in 2016 in Hong Kong were summarised in Supplementary Table S2 and S3, respectively. The age-specific incidence and prevalence of gout in both sexes increased in a non-linear fashion. 6.26% of the general population aged 80 years or older had gout in 2016. The incidence and prevalence of gout in male were higher than in female across all age groups. However, both the incidence ratio and prevalence ratio decreased with increasing age.

**Fig. 3:**
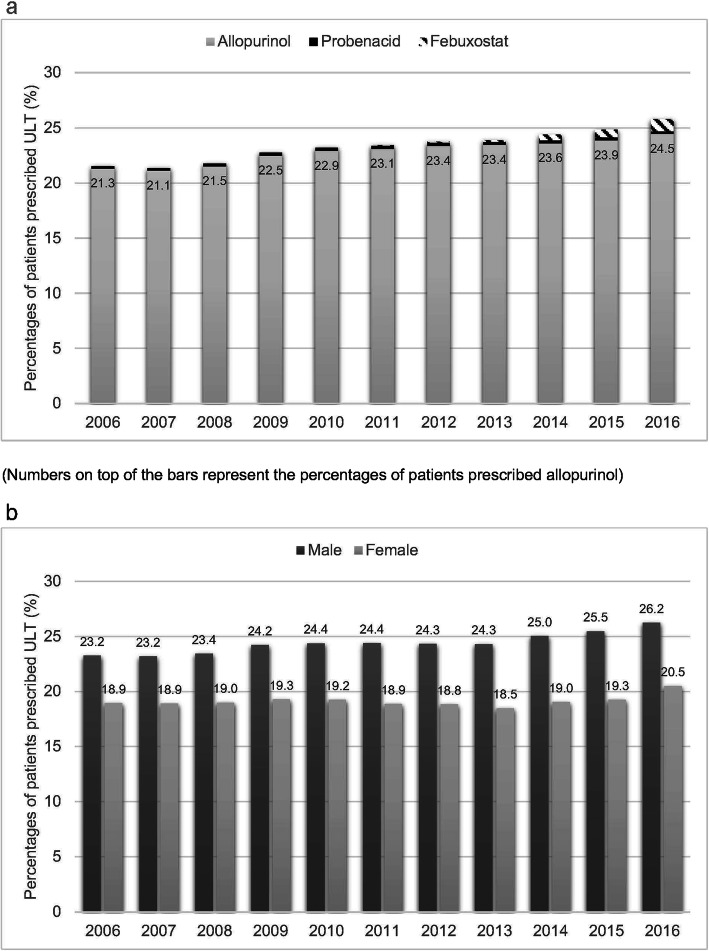
**a** Use of ULT in Hong Kong from 2006 to 2016. **b** Use of ULT stratified by sex from 2006 to 2016

Despite the increase in incidence and prevalence of gout in Hong Kong, the utilisation of ULT remained low. In 2016, only 25.5% of patients with gout were prescribed ULT. Female patients with gout were less often prescribed ULT than male patients. The most commonly prescribed ULT was allopurinol, and its average doses are summarised in Table 1. Among patients treated with allopurinol, all of them use it for more than 1 year and the mean duration of allopurinol therapy was 5 years. However, the mean time lag between diagnosis and treatment was 2.18 [2.17–2.19] years. Less than 1% of patients were prescribed febuxostat or probenecid (Fig. 3).

**Table 1:** Mean doses of allopurinol prescribed for patients with gout from 2006 to 2016

Year	Mean doses of allopurinol [95% CI]		
	Male	Female	Overall
2006	145.03 [143.39–146.68]	126.13 [124.18–128.12]	139.19 [137.90–140.50]
2007	145.17 [143.55–126.13]	126.13 [124.20–128.08]	139.34 [138.05–140.63]
2008	147.00 [145.41–126.07]	126.07 [124.18–127.99]	140.59 [139.33–141.86]
2009	148.60 [147.06–150.15]	125.90 [124.09–127.75]	141.71 [140.49–142.94]
2010	150.15 [148.67–151.65]	126.79 [125.02–128.60]	143.09 [141.91–144.28]
2011	151.78 [150.33–153.24]	128.05 [126.28–129.85]	144.69 [143.53–145.85]
2012	153.19 [151.77–154.64]	129.67 [127.93–131.43]	146.13 [144.99–147.28]
2013	154.27 [152.85–155.70]	129.41 [127.69–131.15]	146.81 [145.68–147.95]
2014	154.57 [153.18–155.97]	129.67 [127.98–131.38]	147.10 [145.99–148.22]
2015	154.34 [152.98–155.71]	128.14 [126.50–129.80]	146.43 [145.35–147.53]
2016	153.55 [152.24–154.87]	127.44 [125.85–129.05]	145.66 [144.61–146.71]

*95% CI* 95% confidence interval

**Fig. 4:**
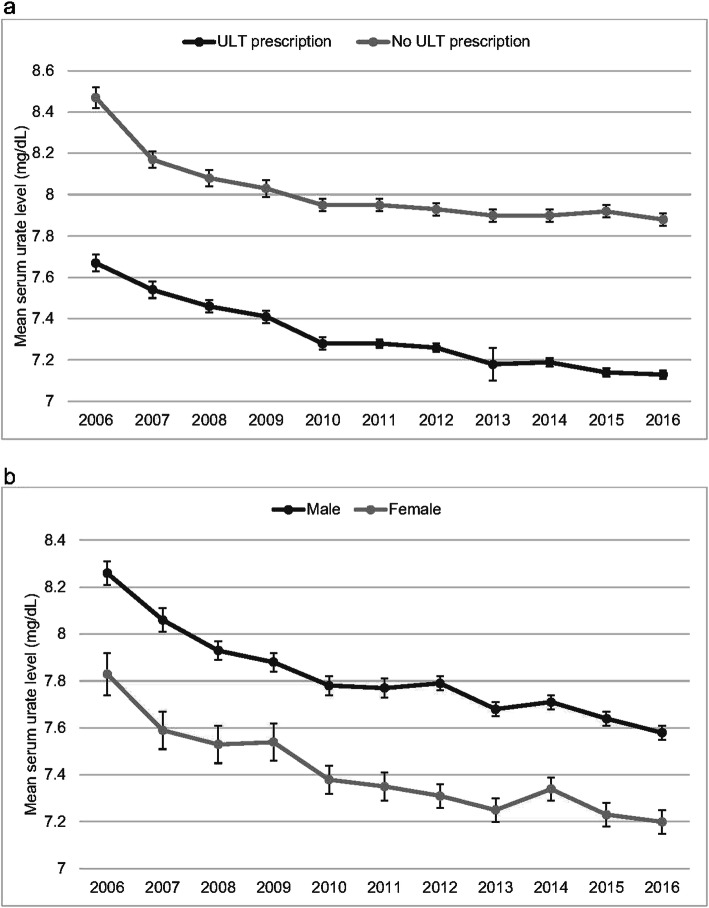
**a** Mean serum urate levels in patients with gout from 2006 to 2016. **b** Mean serum urate levels in patients with gout stratified by sex

**Fig. 5:**
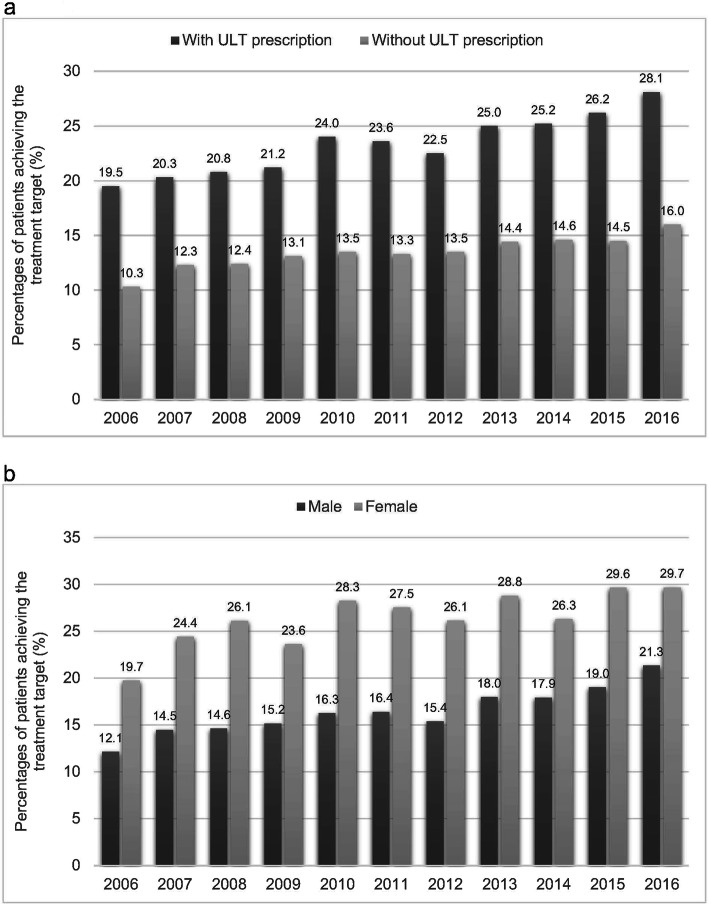
**a** Percentages of patients with gout who can achieve the treatment target (< 6 mg/dL) from 2006 to 2016. **b** Percentages of patients with gout who can achieve the treatment target (< 6 mg/dL) stratified by sex

There was a steady decrease in the mean serum urate levels in patients with gout from 2006 to 2016 as illustrated in Fig. 4. Female patients and patients treated with ULT had lower serum urate levels. In 2016, the mean serum urate levels in patients with or without ULT prescriptions were 7.88 mg/dL and 7.13 mg/dL respectively. The estimated proportions of patients who were able to achieve the treatment target (< 6 mg/dL) are illustrated in Fig. 5. In general, patients treated with ULT were more likely to achieve the treatment target and the percentage also increased from 19.5 to 28.1% from 2006 to 2016. Although less female patients were prescribed ULT, they were more likely to achieve the treatment target than male patients.

## Discussion

This is the latest population-based study that investigates the incidence and prevalence of gout in Hong Kong. Subjects included in this study are representative of the general population. We reported an increase in the incidence and prevalence of gout in Hong Kong. Ageing population is definitely one of the contributing factors. The number of Hong Kong people aged ≥ 65 years increased from 865,200 (12.5%) in 2006 to 1,192,700 (16.1%) in 2015 [14]. As population ageing continues, we expected the crude incidence and prevalence of gout will further increase.

Our study also showed a non-linear increase in age-specific incidence and prevalence of gout. This phenomenon was also shown in a population-based study in Taiwan [4]. The incidence of gout was highest at the age ≥ 80 because this age group had the highest number of incident cases but the lowest PY.

The latest incidence and prevalence of gout in Hong Kong were similar to those in the western countries, including the UK [2, 15], New Zealand [16], Denmark [17], Australia [18], and the US according to the US National Health Nutrition and Examination Survey in 2015–2016 [18]. However, the prevalence of gout did not increase in the US.

An increase in both the age- and sex-adjusted incidence and prevalence suggested that other risk factors, including alcohol consumption, obesity, and diabetes, might contribute to this observation. The Population Health Survey showed that the proportion of Hong Kong people with overweight or obesity increased from 38.8% in 2003/2004 to 50.0% in 2014/2015 [14, 19]. Results of this survey also showed that there was an increase in regular alcohol consumption. The proportion of population with regular alcohol consumption increased from 9.5% in 2003/2004 to 11.1% in 2013/2014, while the prevalence of self-reported diabetes increased from 3.8 to 5.5%. Although CDARS does not capture body mass index and alcohol consumption, results of this survey may explain the increase in the incidence and prevalence of gout beyond population ageing.

The management of gout remained suboptimal in Hong Kong because the utilisation of ULT was insufficient [4]. The prescription of febuxostat was less than 1% because it was not subsidised by the government. Although allopurinol was fully subsidised, only 24.5% of patients with gout received this medication [12].

The efficacy of ULT in reducing serum urate levels has been demonstrated in many randomised controlled trials. In addition, the use of xanthine oxidase inhibitors in patients with gout is associated with cardiovascular and renal benefits. Epidemiological studies suggested that allopurinol might decrease mortality in patients with congestive heart failure and the risk of myocardial infarction [20]. However, allopurinol is associated with an increased risk of severe cutaneous adverse reaction in patients carrying the HLA-B*5801 allele. According to a population-based study conducted in Taiwan, among Han Chinese who carry the HLA-B*5801 allele are 580 times more likely to develop allopurinol-induced severe cutaneous adverse reaction than those who do not carry the allele [21]. Although febuxostat is more effective than allopurinol, it is associated with increased cardiovascular and all-cause mortality compared to allopurinol [22]. It is worth noting that the results have been criticised due to high drop-out and many other methodological issues. Nevertheless, many studies have confirmed that hyperuricaemia is associated with increased mortality, and therefore, ULT should be considered seriously in patients with gout.

### Limitation

This study is not without limitations. We used the diagnosis codes in CDARS; therefore, the diagnostic accuracy depends on physician’s coding. Compliance to ULT cannot be captured in CDARS. Therefore, the effect of compliance to ULT on serum urate levels among patients with gout cannot be assessed in this study. In addition, not all patients with gout had a serum urate level measurement. Therefore, the mean serum urate levels cannot represent all the patients with gout in this study. Although we covered more than 90% of the general population in Hong Kong, CDARS does not include patients receiving medical care in the private sector. This social bias could not be remedied as socio-economic data are not included in the database.

## Conclusion

Population ageing contributes to an increase in the incidence and prevalence of gout in Hong Kong. The crude prevalence of gout in Hong Kong was 2.92% in 2016, which is comparable to that reported in other western countries. Despite an increase in the prevalence of gout, the utilisation of ULT remained low. Only one in four patients with gout was prescribed urate-lowering agents.

## Supplementary information

**Additional file 1: Supplementary Fig. S1.** Subject inclusion.

**Additional file 2: Supplementary Table S1.** Age- and sex-adjusted incidence and prevalence of gout in Hong Kong from 2006 to 2016. **Supplementary Table S2.** Age- and sex-specific incidence of gout in Hong Kong in 2016. **Supplementary Table S3.** Age- and sex-specific prevalence of gout in Hong Kong in 2016.

## Data Availability

The datasets used and analysed during the current study are available from the corresponding author on reasonable request.

## Abbreviations

US: United States
ACR: American College of Rheumatology
EULAR: European League Against Rheumatism
ULT: Urate-lowering therapy
HA: Hospital Authority
CDARS: Clinical Data Analysis and Reporting System
PY: Person-years
CI: Confidence interval
