# Supplementary material for: Epidemiology of gout in Hong Kong: a population-based study from 2006 to 2016
Source: Arthritis Res Ther. 2020 Sep 4;22:204. doi: 10.1186/s13075-020-02299-5 (PMC7487938; doi:10.1186/s13075-020-02299-5)
Supplement: Supplementary file 1 — Additional file 1: Supplementary Fig. S1. Subject inclusion. [file 13075_2020_2299_MOESM1_ESM.pdf]

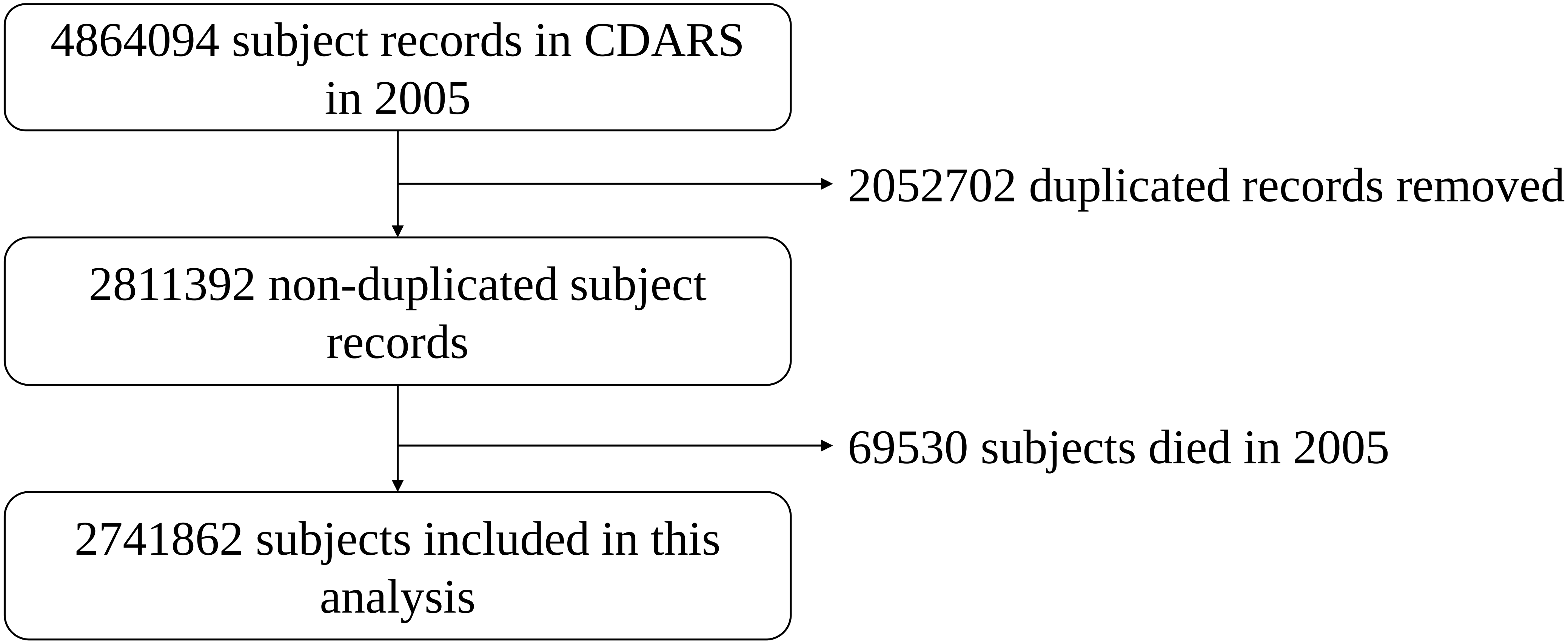
4864094 subject records in CDARS in 2005
2052702 duplicated records removed
2811392 non-duplicated subject records
69530 subjects died in 2005
2741862 subjects included in this analysis
