# Supplementary material for: Epidemiology of gout in Hong Kong: a population-based study from 2006 to 2016
Source: Arthritis Res Ther. 2020 Sep 4;22:204. doi: 10.1186/s13075-020-02299-5 (PMC7487938; doi:10.1186/s13075-020-02299-5)
Supplement: Supplementary file 2 — Additional file 2: Supplementary Table S1. Age- and sex-adjusted incidence and prevalence of gout in Hong Kong from 2006 to 2016. Supplementary Table S2. Age- and sex-specific incidence of gout in Hong Kong in 2016. Supplementary Table S3. Age- and sex-specific prevalence of gout in Hong Kong in 2016. [file 13075_2020_2299_MOESM2_ESM.docx]

Supplementary Table S1. Age- and sex-adjusted incidence and prevalence of gout in Hong Kong from 2006-2016

| Year | Age- and sex-adjusted incidence [95% CI] (/100000 PY) | Age- and sex-adjusted prevalence [95% CI] (%) |
| --- | --- | --- |
| 2006 | 99.41 [97.70-101.12] | 1.08 [1.06-1.10] |
| 2007 | 102.94 [101.23-104.65] | 1.14 [1.12-1.15] |
| 2008 | 119.54 [117.76-121.33] | 1.22 [1.20-1.24] |
| 2009 | 140.93 [138.86-143.00] | 1.31 [1.29-1.33] |
| 2010 | 183.02 [180.49-185.56] | 1.43 [1.41-1.46] |
| 2011 | 168.83 [166.48-171.19] | 1.53 [1.50-1.55] |
| 2012 | 169.56 [167.14-171.98] | 1.60 [1.58-1.63] |
| 2013 | 173.35 [170.49-176.21] | 1.62 [1.60-1.65] |
| 2014 | 205.57 [201.67-209.46] | 1.73 [1.70-1.76] |
| 2015 | 238.57 [232.85-244.30] | 1.79 [1.76-1.82] |
| 2016 | 294.74 [285.67-303.80] | 1.84 [1.81-1.86] |

Abbreviations used in this table: 95% CI: 95% confidence interval; PY: person-years.

Supplementary Table S2. Age- and sex-specific incidence of gout on Hong Kong in 2016

| Age groups | Age-specific incidence [95% CI] (/100000 PY) | | | Male-to-female incidence rate ratio [95% CI] |
| --- | --- | --- | --- | --- |
|  | Male | Female | Total |  |
| 20-29 | 38.72 [28.40-49.05] | 4.14 [0.51-7.77] | 22.68 [16.89-28.46] | 9.35 [2.75-31.75] |
| 30-39 | 71.84 [56.74-86.93] | 2.46 [0.30-4.62] | 33.77 [26.87-40.67] | 29.20 [11.37-74.99] |
| 40-49 | 83.53 [69.84-97.22] | 9.35 [5.61-13.10] | 51.49 [43.68-59.3] | 8.93 [5.02-15.90] |
| 50-59 | 174.82 [154.63-195.01] | 45.40 [36.76-54.05] | 92.12 [83.03-101.22] | 3.85 [2.54-5.84] |
| 60-69 | 497.85 [454.52-541.19] | 173.19 [150.04-196.34] | 181.20 [167.98-194.42] | 2.87 [1.80-4.59] |
| 70-79 | 774.42 [709.22-839.62] | 348.42 [312.63-384.22] | 400.39 [374.32-426.47] | 2.22 [1.42-3.48] |
| 80 or above | 5346.25 [4956.01-5736.49] | 2782.39 [2600-2964.78] | 734.48 [698.66-770.3] | 1.92 [1.02-3.63] |

Abbreviations used in this table: 95% CI: 95% confidence interval; PY: person-years.

Supplementary S3. Age- and sex-specific prevalence of gout on Hong Kong in 2016

| Age groups | Age- specific prevalence [95% CI] (%) | | | Male-to-female prevalence ratio [95% CI] |
| --- | --- | --- | --- | --- |
|  | Male | Female | Total |  |
| 20-29 | 0.19 [0.17-0.22] | 0.03 [0.02-0.04] | 0.12 [0.1-0.13] | 7.27 [7.24-7.29] |
| 30-39 | 0.84 [0.79-0.89] | 0.1 [0.08-0.11] | 0.43 [0.41-0.46] | 8.82 [8.77-8.88] |
| 40-49 | 2.16 [2.08-2.24] | 0.25 [0.23-0.28] | 0.98 [0.94-1.01] | 8.51 [8.43-8.6] |
| 50-59 | 3.43 [3.35-3.52] | 0.64 [0.61-0.67] | 1.77 [1.74-1.81] | 5.38 [5.29-5.47] |
| 60-69 | 5.39 [5.29-5.50] | 1.51 [1.46-1.56] | 3.16 [3.10-3.21] | 3.57 [3.46-3.69] |
| 70-79 | 8.03 [7.87-8.19] | 3.56 [3.46-3.66] | 5.63 [5.54-5.72] | 2.26 [2.07-2.45] |
| 80 or above | 13.34 [13.13-13.55] | 6.26 [6.07-6.45] | 9.99 [9.88-10.11] | 2.13 [1.85-2.41] |

Abbreviations used in this table: 95% CI: 95% confidence interval; PY: person-years.
